# Supplementary figures and images for: Comparative analysis of the caecal tonsil transcriptome in two chicken lines experimentally infected with Salmonella Enteritidis
Source: PLoS One. 2022 Aug 17;17(8):e0270012. doi: 10.1371/journal.pone.0270012 (PMC9384989; doi:10.1371/journal.pone.0270012)

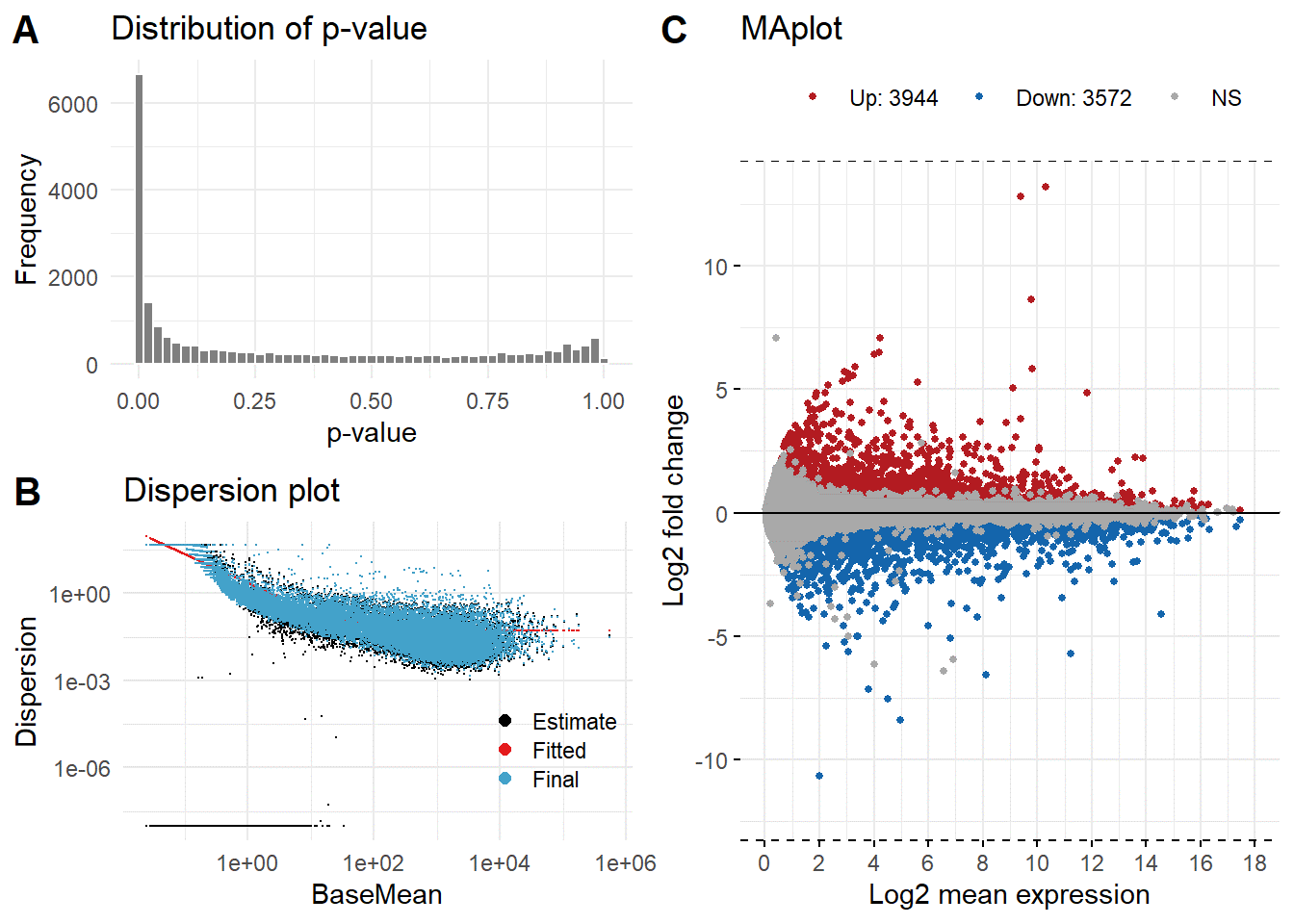

Supplement: S1 Fig — Distribution of raw p-values (A), dispersion plot (B), and MA plot highlighting 1492 and 1285 up- and down-regulated genes with a p-adj < 0.05 (C). (TIFF) [file pone.0270012.s004.tiff]

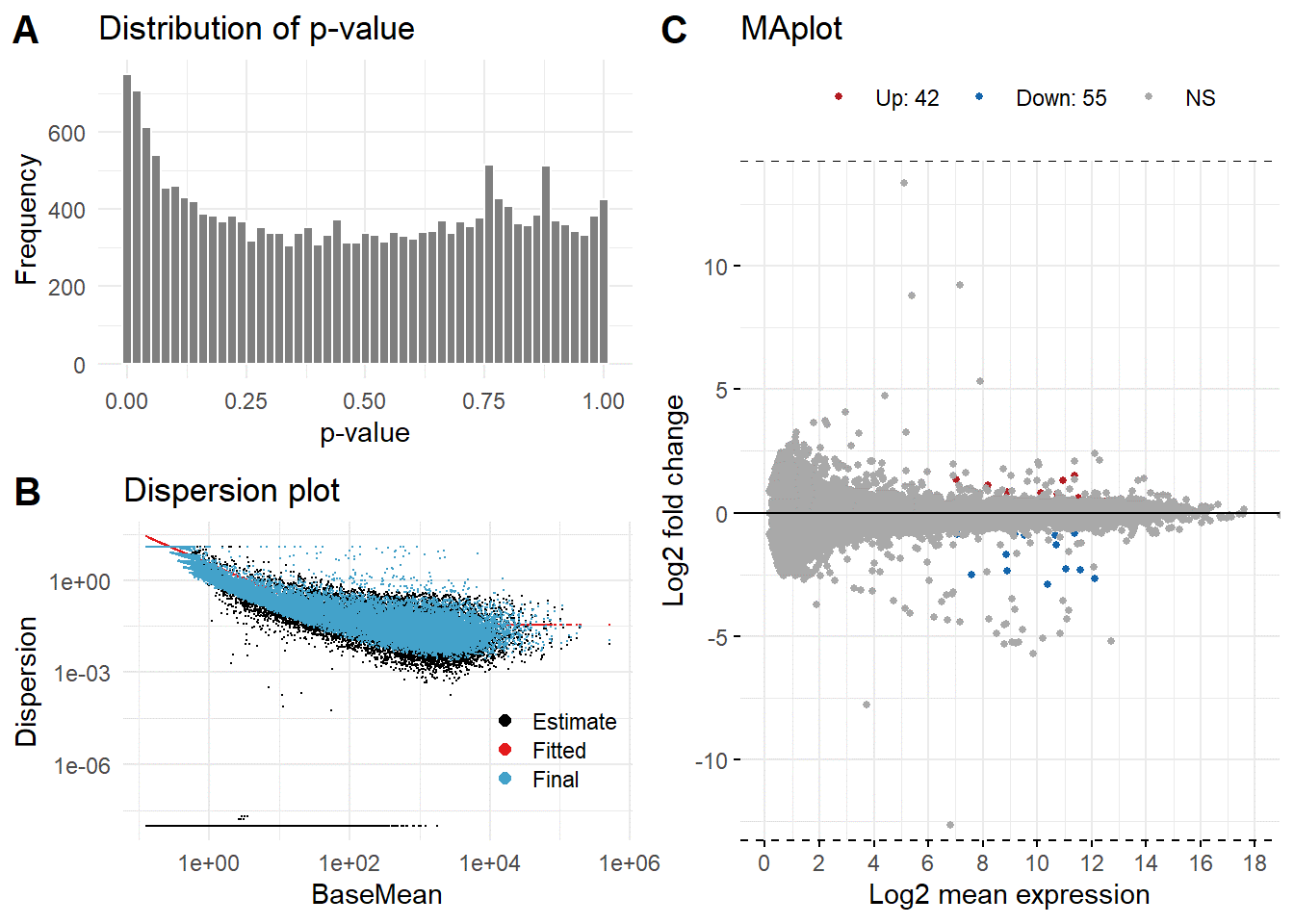

Supplement: S2 Fig — Distribution of raw p-values (A), dispersion plot (B), and MA plot highlighting 42 and 55 up- and down-regulated genes with a p-adj < 0.05 (C). (TIFF) [file pone.0270012.s005.tiff]

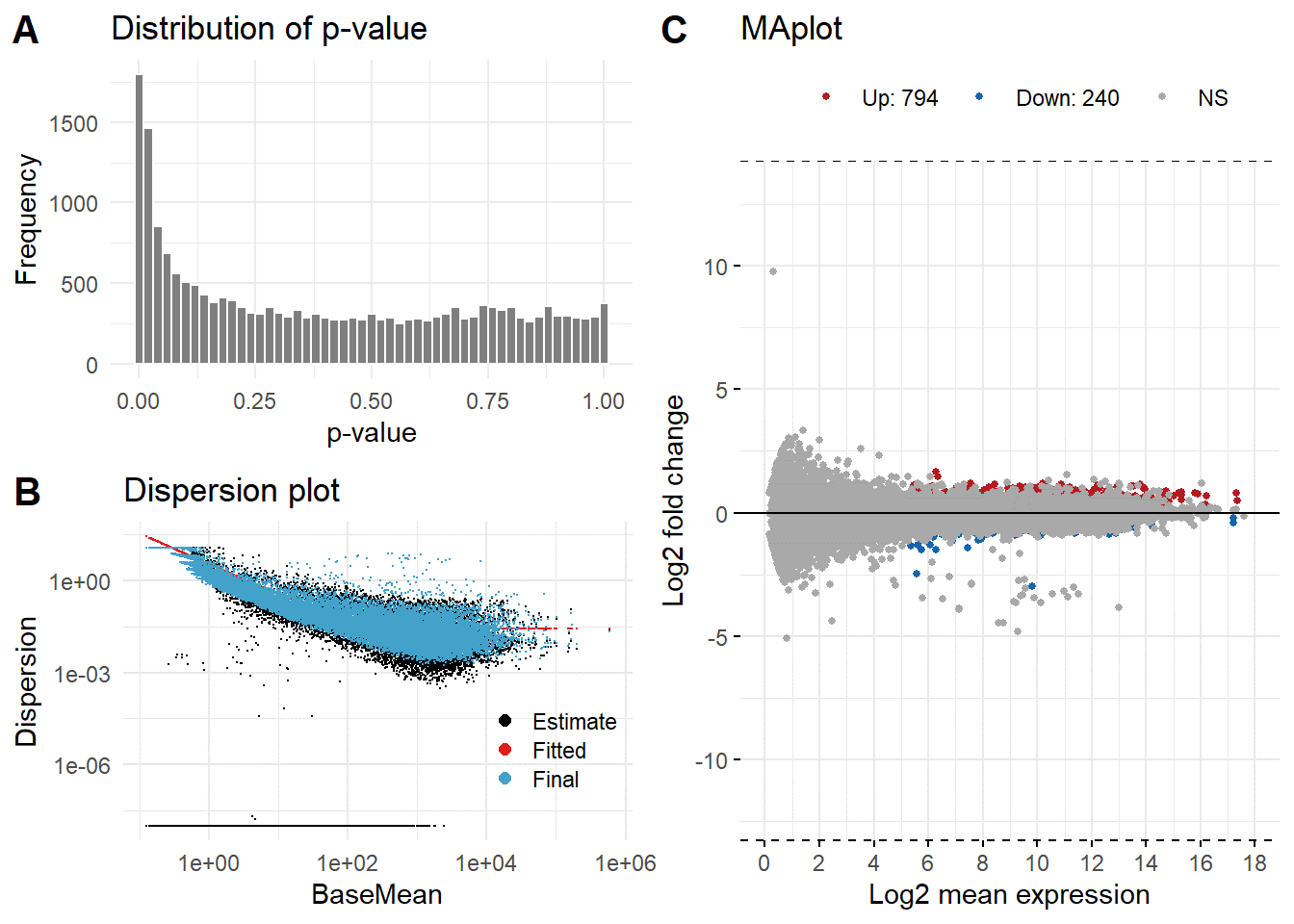

Supplement: S3 Fig — Distribution of raw p-values (A), dispersion plot (B), and MA plot highlighting 794 and 240 up- and down-regulated genes with a p-adj < 0.05 (C). (TIFF) [file pone.0270012.s006.tiff]
